# False Teeth in Œsophagus Fifteen Days

**Published:** 1860-02

**Authors:** A. P. Dutcher


### FALSE TEETH IN ŒSOPHAGUS FIFTEEN DAYS.

BY A. P. DUTCHER, M. D.

Mrs. S., aged twenty-five, just after retiring to rest last evening had an epileptic convulsion, after which she was insensible during the remainder of the night. In the morning she was sick at the stomach, and vomited very freely several times. In the afternoon the sickness left her, and she tried to take some food, but was unable to swallow. The next morning she could swallow water, but nothing in the form of solid aliment. Having now fully recovered her senses, she for the first time informed her friends that she had swallowed her false teeth. I was then called in, and on examination found them lodged in the oesophagus just above the diaphragm. As I had no throat forceps of sufficient length to reach them, and did not like to force them into the stomach, I told the family they had better call in some surgeon, who would have the necessary instruments for their removal. As they did not cause her any pain, she thought that on the next day she would go to Salem, Columbiana county, Ohio, and have them taken out. She did not, however, go until Mondav, the 31st. The individual that was applied to had forceps, but none long enough to reach them. He concluded, however, with the consent of Mrs. S., to pass them into the stomach. After manipulating for a few minutes with a gum-tube, he assured her that they were in the stomach. She returned home the same evening. Her throat was not much inflamed from the operation. She thought she could swallow some better. In other respects she was very comfortable.

On Friday morning, Nov. 4, she took labor, and had a speedy delivery of a child at term. I did not attend her during the confinement. She appeared to do very well until Thursday, the 10th, when I was again called to see her. She was still unable to swallow anything but fluids. Pulse 90, and respiration 25 per minute. Complained of pain at the inferior portion of the sternum, and under the right scapula. From the pain and difficulty of swallowing, I came to the conclusion that the teeth were still in the oesophagus. On examination, I found I was correct. The case now having assumed an alarming character, Dr. John Dickson, of Pittsburgh, Pa., was sent for. He eame on Friday afternoon. After the most persevering efforts and skillful manipulation, he succeeded in extricating them from the position they had occupied for more than fifteen days. Her throat was very sore for several days; had some fever, cough, and expectoration, but is now (Dec. 2) in her usual health.

The plate was made of silver. It was two inches long and one inch wide, with four teeth upon it. The instruments used in extricating it were a probang and the long throat-forceps. The probang was such as is commonly used in cauterising the throat. A piece of muslin was tightly drawn over the sponge, and secured to the handle; it was then thickly covered with nooses of strong thread. The forceps were fourteen inches in length, and made after the fashion of Weiss' urethra-forceps, used by many surgeons for the removal of calculi under a certain size from the bladder.

*Removal of Body of Superior Maxillary Bone.*—By the courtesy of Prof. Pancoast, the operation for removal of the superior maxillary bone was performed by his colleague, Dr. Gross. The patient, was 69 years of age, a miller, and of temperate habits. The tumor protruded outwardly, and also toward the nasal cavity. It had gradually increased since the time it was observed, four months ago.

The affection Dr. G. presumed to be encephaloid—fungus hæmatodes—soft cancer, the nature and structure of which it was unnecessary now to dilate upon, as he had lately explained it fully in his lectures. The disease here had evidently commenced in the maxillary sinus, probably in the mucous membrane, possibly in its periosteal lining. It was comparatively limited. The alveolar and palate processes were not affected; only the anterior wall of the antrum was involved. It was without pain, there was no discharge, and the general health of the patient was good. It was, therefore, a case in which the surgeon was justifiable in performing an operation for the relief of the patient.

If the tumor should remain unmolested, it would encroach, ere long, upon the surrounding structures in all directions. At a later stage we should find involvement of the lymphatic ganglions in front of the ear and below the jaw. When the disease was in this stage he usually declined operating, as he should also when it made rapid progress, or was of long standing, with marked cancerous cachexia in the patient, evidenced by sallow complexion and rapid wasting of the flesh and strength.

The operation would consist in the excision of the diseased structure, cutting freely into the surrounding structures, so as to remove the atmosphere, as it were, which enveloped the diseased portion. The data of operations upon the upper jaw, Dr. G. remarked, were imperfect. He had performed many, not always with a happy result. His colleague, Dr. Pancoast, he believed, had performed more, and met with greater success. It was certain, so far as his experience went, that the operation was not so generally successful as that upon the lower jaw. He must say to this patient that he would perform the operation to the best of his ability, and leave the result to nature.

After the administration of ether and the extraction of the middle incisor tooth of the diseased side and a molar, which were in the way of the operation, a curvilinear incision was made from the ear to the middle of the right side of the upper lip. The hemorrhage following this incision made it necessary to stop and secure several bleeding vessels. The cheek was then separated from the bone, and the outside of the tumor exposed. At its outer limit the union was detached by sawing through the malar bone; the ascending process and horizontal portion of the superior maxillary were divided by pliers, and the diseased body of the bone was removed in mass. A portion of the orbital surface was also included. The excavation was then examined with minuteness, in order to remove every portion of the cancerous growth. The wound, Dr. G. observed, would be closed with the twisted suture, and he would expect it to heal by the first intention.—*Phila. Med. and Surg. Reporter.*

